# Factors Determining the Behavioral Intention to Use Mobile Learning: An Application and Extension of the UTAUT Model

**DOI:** 10.3389/fpsyg.2019.01652

**Published:** 2019-07-16

**Authors:** Cheng-Min Chao

**Affiliations:** Department of Business Administration, National Taichung University of Science and Technology, Taichung, Taiwan

**Keywords:** mobile learning, mobile self-efficacy, unified theory of acceptance and use of technology model, trust, perceived enjoyment, perceived risk

## Abstract

This study developed and empirically tested a model to predict the factors affecting students’ behavioral intentions toward using mobile learning (m-learning). This study explored the behavioral intention to use m-learning from the perspective of consumers by applying the extended unified theory of acceptance and use of technology (UTAUT) model with the addition of perceived enjoyment, mobile self-efficacy, satisfaction, trust, and perceived risk moderators. A cross-sectional study was conducted by employing a research model based on multiple technology acceptance theories. Data were derived from an online survey with 1,562 respondents and analyzed using structural equation modeling. Partial least squares (PLS) regression was used for model and hypothesis testing. The results revealed that (1) behavioral intention was significantly and positively influenced by satisfaction, trust, performance expectancy, and effort expectancy; (2) perceived enjoyment, performance expectancy, and effort expectancy had positive associations with behavioral intention; (3) mobile self-efficacy had a significantly positive effect on perceived enjoyment; and (4) perceived risk had a significantly negative moderating effect on the relationship between performance expectancy and behavioral intention. Our findings correspond with the UTAUT model and provide a practical reference for educational institutions and decision-makers involved in designing m-learning for implementation in universities.

## Introduction

With the recent rapid advancement in mobile telecommunication technologies, mobile phone applications have changed not only how we use mobile phones but also our lives. People now through new methods by using mobile gadgets and technologies. Thus, mobile devices are a crucial tool for mobile health, banking, and mobile learning (m-learning) (Alalwan et al., 2017; Briz-Ponce et al., 2017; Hoque and Sorwar, 2017; Nikou and Economides, 2017; Crompton and Burke, 2018). M-learning is a tool with considerable potential that provides new possibilities for education and learning assessment (Nikou and Economides, 2017). The United Nations Educational, Scientific and Cultural Organization (UNESCO) indicated the potential of m-learning to enhance learning quality and students’ test results. In addition, UNESCO has suggested that governments should adopt new technologies to secure equal access to mobile connectivity and enable students to gain further learning possibilities (UNESCO, 2009). M-learning is a critical component of higher education, and thus its acceptance and adoption receives growing interest. However, recent studies (Kim et al., 2017; Hamidi and Chavoshi, 2018) have indicated that although many universities have extended their online learning platforms to mobile services, students’ interest and usage of m-learning is not as high as expected. Thus, investigating the factors affecting university students’ acceptance of m-learning and their intentions to use it in a comprehensive and integrated manner is critical (Nikou and Economides, 2017; Briz-Ponce et al., 2017). Therefore, this study examined the behavioral intentions of university students to use m-learning.

Effective implementation of any information technology (IT) or information system (IS) depends on user acceptance (Davis, 1989). In recent decades in the domains of psychology, ISs, and sociology, numerous theoretical models have been developed to predict and explain user acceptance of IT or ISs. One of the most widely cited frameworks in the field of IT and ISs is the technology acceptance model (TAM) (Chauhan and Jaiswal, 2016; Cimperman et al., 2016; Šumak and Šorgo, 2016; Šumak et al., 2017). However, some scholars (Sánchez-Prieto et al., 2016; Šumak et al., 2017; Tsai et al., 2018) have contended that the TAM has several disadvantages, including (1) not providing adequate insight into individuals’ perspectives of novel systems; (2) neglecting its indicators and directly investigating the external variables of perceived ease of use (PEOU) and perceived usefulness (PU); and (3) ignoring the relationship between usage attitude and usage intention. In their search for a more complete IT acceptance model and to address the weaknesses of the TAM, Venkatesh et al. (2003) integrated core elements from eight models and prominent theories (including the theory of reasoned action [TRA], innovation diffusion theory [IDT], the theory of planned behavior [TPB], the TAM; the combined TAM-TPB, the motivational model (MM), the model of PC utilization [MPCU], and social cognitive theory [SCT]) to predict or explaining new technology adoption, acceptance, and usage, and proposed a unified model called the unified theory of acceptance and use of technology (UTAUT) model.

Since its introduction, the UTAUT model has been applied and tested extensively for predicting system usage and making technology-adoption- and technology-usage-related decisions in various fields such as interactive whiteboards (Šumak and Šorgo, 2016; Šumak et al., 2017), near-field communication technology (Khalilzadeh et al., 2017), mobile health (Hoque and Sorwar, 2017), home telehealth services (Cimperman et al., 2016), and acceptance of Enterprise Resource Planning (ERP) software (Chauhan and Jaiswal, 2016). Applied research regarding the UTAUT model has been extensive. This model provides a framework that not only explains acceptance of IT and ISs but also elucidates the actual use of such technologies and systems. Because of its capability to integrate different the TAMs, the UTAUT model contributes substantially to the exploration of technology acceptance and usage (Venkatesh et al., 2003). Therefore, this study used the UTAUT model as the theoretical basis to evaluate the influences of technology-related factors on m-learning adoption.

Although the UTAUT model has been widely adopted, doubts exist over its capability to explain individuals’ technology acceptance. Thus, the original UTAUT model has been extended. Many researchers (Martins et al., 2014; Maillet et al., 2015; Cimperman et al., 2016; Kabra et al., 2017; Khalilzadeh et al., 2017) have suggested that increasing the number of external variables can enhance this model’s ability to predict the acceptance of IT. Several variables have been recommended to complement the original UTAUT model (e.g., self-efficacy, trust, habits, satisfaction, and perceived risk). For example, Kabra et al. (2017) incorporated personal innovation specific to IT and trust into the UTAUT model to evaluate the factors that influence users’ behavioral intentions to use IT. Khalilzadeh et al. (2017) included self-efficacy, risk, trust, security, and attitude to evaluate the factors that influence users’ behavioral intentions to make mobile payments. According to previous study on mobile technologies (Alalwan et al., 2017; Khalilzadeh et al., 2017), trust is a crucial factor determining users’ behavioral intentions to adopt technology. Chang et al. (2017) posited that perceived enjoyment is critical in explaining e-learning adoption. As mentioned, the present study proposed an extension of the UTAUT model by adding variables (mobile self-efficacy, perceived enjoyment, satisfaction, perceived risk, and trust) to predict adoption of m-learning.

The UTAUT model was adopted and extended by incorporating the constructs of mobile self-efficacy and perceived enjoyment in addition to security-related constructs (i.e., satisfaction, trust, and perceived risk) to investigate university students’ behavioral intentions toward using m-learning in higher education. The UTAUT model was modified by incorporating new constructs such as perceived enjoyment, mobile self-efficacy, satisfaction, trust, and perceived risk. The modified model was then empirically tested. The four primary objectives of this study were (1) to investigate the factors influencing behavioral intention to use m-learning in education; (2) to develop an extended UTAUT model incorporating perceived enjoyment, mobile self-efficacy, trust, satisfaction, and perceived risk for m-learning; (3) to examine whether effort expectancy, performance expectancy, and perceived risk moderate and predict behavioral intention to use m-learning; and (4) to assess the resultant model empirically. To achieve the aforementioned objectives, the following research questions were formulated. (1) What factors determine students’ behavioral intentions to use m-learning for educational purposes? (2) Do perceived enjoyment, mobile self-efficacy, trust, and satisfaction affect the UTAUT model in relation to m-learning? (3) Does mobile self-efficacy influence perceived enjoyment in m-learning? (4) How does perceived risk moderate the effects of effort expectancy and performance expectancy on behavioral intention to use m-learning? This research is expected to contribute to the literature by (1) identifying satisfaction, trust, and perceived enjoyment as antecedents of m-learning usage; (2) advancing the theoretical understanding of behavioral intention among university students with respect to m-learning; (3) providing empirical evidence of the effects of external factors on effort expectancy and performance expectancy, which lead to usage-related satisfaction and behavioral intention; (4) proving that perceived risk moderates the effects of effort and performance expectancy; (5) providing a reference for teachers and educational institutions for deciding future development directions and approaches related to the implementation of m-learning.

## Literature Review and Hypothesis Development

The hypotheses developed in the current study were based on a robust foundation derived from contemporary studies. To achieve the research objectives, four external variables (mobile self-efficacy, perceived enjoyment, satisfaction, and trust) were used as external variables for the proposed UTAUT model. This study employed and empirically tested the proposed UTAUT model in the context of m-learning by recruiting university students in central Taiwan and determining the effects of the four aforementioned external variables on students’ effort expectancy, performance expectancy, and satisfaction toward m-learning. This study determined how students and their behavioral intention toward m-learning can be influenced by their attitude. Perceived risk was considered to have had a moderating effect on the interrelationships between effort expectancy, performance expectancy, and behavioral intention.

### Definition of M-Learning

The rapid advancement of mobile and wireless technologies has resulted in increasing use of mobile devices in education and has changed approaches to learning. Additionally, new terms such as e-learning and m-learning have been coined. Over the preceding 10 years, use of IT has expanded from programmed instruction, through computer-assisted instruction, to Internet-connected e-learning, and further to m-learning. In particular, m-learning for educational use has become increasingly common, and thus has received increasing attention from researchers and educators (Briz-Ponce et al., 2017; Kim et al., 2017; Nikou and Economides, 2017; Crompton and Burke, 2018; Hamidi and Chavoshi, 2018; Hamidi and Jahanshaheefard, 2019). M-learning is a critical component of higher education that enables students to learn anytime and anywhere. However, although m-learning is a pertinent topic of discussion, a single definition has not been established. Hamidi and Chavoshi (2018) argued that alongside the Internet and the development of technology, m-learning offers an online learning environment through which students can learn and interact. Martin and Ertzberger (2013) defined m-learning as a method of learning that is enabled when learners have access to information anytime and anywhere through mobile technologies, allowing them to participate in authentic activities while learning. Yousafzai et al. (2016) defined m-learning as a learning process where learners are not restrained by fixed locations and can benefit from access to learning materials through mobile devices. Similar to other teaching methods, m-learning has many advantages from the perspective of users, such as a substantial amount of learning resources, rapid access to information, two-way interaction, and removal of time- and location-related restrictions (Briz-Ponce et al., 2017; Kim et al., 2017; Tang and Hew, 2017; Crompton and Burke, 2018; Hamidi and Chavoshi, 2018; Hamidi and Jahanshaheefard, 2019). In this study, we defined m-learning as a learning process conducted across various contexts (location, time, and other environmental factors) where learners can benefit from access to learning materials through smart mobile devices such as smartphones and tablet computers.

### UTAUT

In the search for a more comprehensive IT acceptance model, Venkatesh et al. (2003) reviewed related studies and conducted an empirical study where they synthesized several elements of the eight behavioral intention models used in previous technology acceptance contexts. These models include (1) the TRA (Sheppard et al., 1988; Davis et al., 1989); (2) the TAM (Davis, 1989; Davis et al., 1989; Venkatesh and Davis, 2000); (3) the TPB (Ajzen, 1991; Taylor and Todd, 1995); (4) the combined TAM-TPB (Taylor and Todd, 1995); (5) the MPCU (Thompson et al., 1991); (6) the MM (Vallerand, 1997); (7) SCT (Bandura, 1986; Compeau and Higgins, 1995); and (8) IDT (Rogers, 2003). Therefore, the researchers applied the UTAUT model to unify the existing theories regarding how users accept technology (Venkatesh and Morris, 2000; Venkatesh et al., 2003).

**FIGURE 1:**
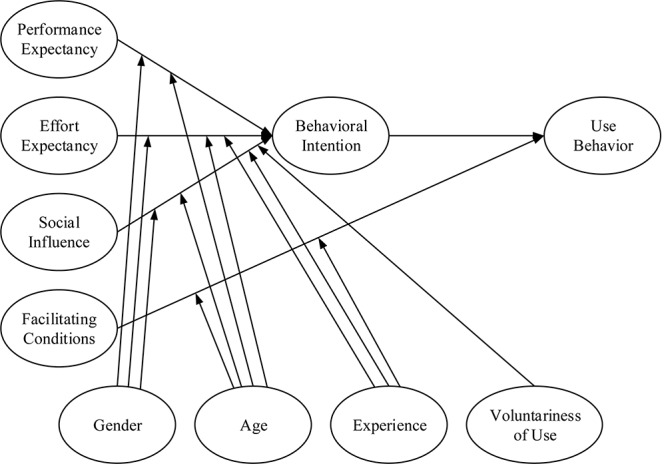
The unified theory of acceptance and use of technology (UTAUT) model.

Based on a systematic analysis and comparison of the aforementioned models, Venkatesh et al. (2003) proposed an integrated model, namely the UTAUT model, which can explain 70% of the variance in user intention. The results of that empirical study demonstrated that the UTAUT model is the most effective model for analyzing technology acceptance. The UTAUT model consists of six main constructs, namely performance expectancy (“PE” hereafter), effort expectancy (“EE” hereafter), social influence (SI), facilitating conditions (FC), behavioral intention (“BI” hereafter) to use the system, and usage behavior (see Figure 1). The UTAUT model contains four essential determining components and four moderators. According to the model, the four determining components of BI and usage behavior are PE, EE, SI, and FC (Venkatesh et al., 2003). Gender, age, experience, and willingness to use are the moderators that affect usage of technology (see Figure 1).

Effort expectancy has been introduced in the UTAUT model, and is a crucial predictor of technology acceptance. According to Venkatesh et al. (2003), EE is “the degree of ease associated with the use of the system.” According to Cimperman et al. (2016), the antecedents of EE are ease of use, complexity, and PEOU. PE has also been introduced in the UTAUT model, and has been defined as “the degree to which an individual believes that the system helps to improve job performance.” BI has been defined as “the degree to which a person has formulated conscious plans regarding whether to perform a specified future behavior.” In the context of the present study, EE represents university students’ beliefs regarding the ease of use of m-learning. PE denotes students’ beliefs regarding whether use of m-learning will enhance their learning performance. Venkatesh et al. (2003) revealed that PE is the strongest determinant of a user’s BI to adopt a technology.

According to one study, (Venkatesh et al., 2003; Šumak and Šorgo, 2016; Hoque and Sorwar, 2017; Khalilzadeh et al., 2017; Šumak et al., 2017) PE and EE are direct determinants of BI. The present study hypothesized that PE and EE can significantly influence students’ BIs toward acceptance and adoption of m-learning. The following hypotheses were proposed.

1. Hypothesis 1: EE has a significant influence on the BIs of university students to use m-learning.
2. Hypothesis 2: PE has a significant influence on the BIs of university students to use m-learning.

### Effects of Satisfaction and Trust

Satisfaction and trust are critical factors for predicting individuals’ BIs toward adopting ISs or IT (Koufaris and Hampton-Sosa, 2004; DeLone and McLean, 2016; Kabra et al., 2017). From the perspective of the IS success model, user satisfaction can significantly influence individuals’ BI to use a particular system (DeLone and McLean, 2016). DeLone and McLean (2016) defined satisfaction as “users’ level of satisfaction with reports, web sites, and support services.” Maillet et al. (2015) indicated that EE and PE had significant effects on satisfaction. In addition, Shiau and Luo (2013) suggested that perceived enjoyment had a significant influence on satisfaction. Therefore, we defined that students’ satisfaction with m-learning may be influenced by not only cognitive appraisals (e.g., EE and PE) but also emotions experience (e.g., perceived enjoyment). In addition, this study argued that students’ satisfaction levels can significantly influence their BIs to use m-learning.

Arpaci (2016) defined trust as “students’ perceptions about the reliability and trustworthiness of the system,” whereas Alalwan et al. (2017) defined it as the “accumulation of trust beliefs: integrity, benevolence, and ability that relate with the bank and mobile-banking channel.” According to previous studies (Arpaci, 2016; Alalwan et al., 2017), students’ trust levels were operationalized as their perceptions of beliefs concerning reliability and trust (i.e., integrity, benevolence, and ability) in relation to m-learning. Notably, research findings regarding the effect of trust on BI remain inconclusive. Although most related studies have identified positive effects of trust on BI, some have found no such relationship (Alalwan et al., 2017; Kabra et al., 2017; Khalilzadeh et al., 2017). For example, Alalwan et al. (2017) confirmed that trust is important in determining users’ likelihood to adopt mobile technologies. The researchers revealed that trust had a considerable effect on students’ BIs toward using m-learning. However, Kabra et al. (2017) found no significant association between trust and BI. We proposed that students’ trust levels positively influence their BIs to use m-learning. Based on this discussion, the following hypotheses were proposed.

1. Hypothesis 3: Satisfaction has a significant influence on the BIs of university students to use m-learning.
2. Hypothesis 4: Trust has a significant influence on the BIs of university students to use m-learning.
3. Hypothesis 5: EE has a significant influence on satisfaction with m-learning.
4. Hypothesis 6: PE has a significant influence on satisfaction with m-learning.
5. Hypothesis 7: Perceived enjoyment has a significant influence on satisfaction with m-learning.

### Effect of Perceived Enjoyment

Perceived enjoyment is a fundamental intrinsic motivation that specifies the extent to which fun can be derived from using IT or an IS. Regarding ISs, Park et al. (2012) defined perceived enjoyment as “the extent to which the activity of using a specific system is perceived to be enjoyable in its own right, aside from any performance consequences resulting from system use.” Accordingly, in the present study, we explored the positive and negative effects of perceived enjoyment on m-learning. The effect of perceived enjoyment on system use was confirmed in a previous study (Sánchez-Prieto et al., 2016; Chang et al., 2017; Tsai et al., 2018), and perceived enjoyment is the most commonly used external factor in the TAM. Perceived enjoyment is a key external factor that significantly influences individuals’ PU, PEOU, and usage intentions toward an IS. However, few studies have examined whether perceived enjoyment is an influential external factor in the UTAUT model. In the UTAUT model, PE and EE are the two most relevant predictors derived from PU and PEOU, which were introduced in the original TAM model (Cimperman et al., 2016). Accordingly, we maintained that perceived enjoyment regarding use of m-learning has significantly positive effects on PE and EE. Based on this discussion, the following hypotheses were proposed.

1. Hypothesis 8: Perceived enjoyment has a significant influence on the EE of m-learning.
2. Hypothesis 9: Perceived enjoyment has a significant influence on the PE of m-learning.

### Effect of Mobile Self-Efficacy

According to SCT proposed by Bandura (1986), self-efficacy refers to people’s assessments of their effectiveness or ability to perform a specific task well; it is related not to the skills of an individual but rather to how he or she utilizes these skills (Bandura, 1986). In this context, self-efficacy is an individual’s personal belief that he or she possesses the aptitude and skills to succeed when engaging in an m-technology-related task (Ozturk et al., 2016). Nikou and Economides (2017) defined mobile self-efficacy as an individual’s perceptions of his or her ability to use mobile devices to accomplish particular tasks (e.g., browsing the Internet). Mobile self-efficacy has been identified as playing a significant role in the adoption of mobile devices to supplement education. To our knowledge, no study has investigated the possible effects of mobile self-efficacy on perceived enjoyment, and theoretical foundations for such a study have not been established. Based on the findings of aforementioned studies, we hypothesized that students’ self-efficacy in using mobile devices can directly affect their perceived enjoyment of m-learning. Therefore, this study proposed the following hypothesis.

1. Hypothesis 10: Mobile self-efficacy has a significant influence on the perceived enjoyment of m-learning.

### Moderating Effect of Perceived Risk

Because this study was investigating the Internet and mobile devices, risk factors in the process of m-learning had to be measured. Users often worry about risks such as privacy problems, system errors, losing passwords, incompatibility of mobile operating systems and security software, and low system quality. Hanafizadeh et al. (2014) stated that risk factors are crucial in mobile services, and the higher the risk of using a new technology, the lower is willingness to use. Alalwan et al. (2018) argued that the likelihood of a customer experiencing a finance- or privacy-related loss during the process in pursuit of a favored consequences of using Internet banking. Featherman and Pavlou (2003) defined perceived risk as the “potential for loss in the pursuit of a desired outcome of using an e-service.” In the present study, we defined perceived risk as the likelihood of a student suffering a loss in the pursuit of m-learning.

To our knowledge, most related studies have examined perceived risk as an external factor influencing the external variables of the UTAUT model (Martins et al., 2014; Alalwan et al., 2018). Alalwan et al. (2018) argued that perceived risk considerably hinders BI. However, no study has examined whether perceived risk acts as moderating factor for any of the UTAUT model’s moderator variables. The present study tested the UTAUT model in relation to m-leaning by adding the factor of perceived risk to the model. We hypothesized that as a moderating factor, perceived risk can influence university students’ EE and PE of m-leaning. In other words, perceived risk moderates the relationships between the independent variables (i.e., EE and PE) and the dependent or outcome variable (i.e., BI). Accordingly, we posited that the relationships between these variables are weakened when perceived risk is considered. To examine this idea in detail, the following moderating effects were hypothesized.

1. M1: The relationship between EE and BI is moderated by perceived risk (“PR” hereafter).
2. M2: The relationship between PE and BI is moderated by PR.

**FIGURE 2:**
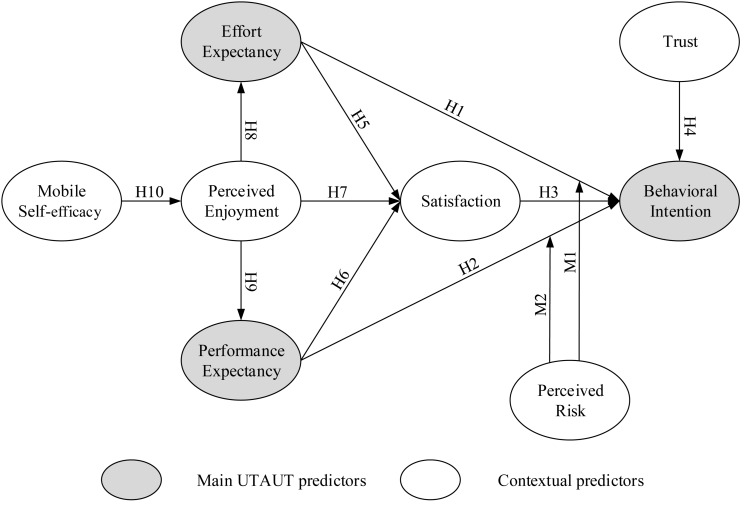
Conceptualized extended UTAUT model for measuring university/college students’ acceptance of mobile learning.

In this study, the UTAUT model was chosen as a basis for investigating university students’ perceptions of m-learning. Figure 2 presents a research model that explains the use of BI for m-learning and the hypothesized relationships between variables. The external UTAUT model variables are grouped based on user factors (mobile self-efficacy, perceived enjoyment, satisfaction, and trust). To analyze the differences in causal relationships among UTAUT factors, we extended the base model by including PR as a variable to assume a moderating role within the model (Figure 2). Eight predictors formed an extended UTAUT model for predicting BI. Figure 2 presents the conceptual model. The relationships among the constructs (arrows) represent the research hypotheses.

## Research Methodology

### Instrumentation and Data Collection Tools

A questionnaire was designed and divided into two sections. In the first section, 31 items were used to measure the eight constructs presented in the research model (Figure 2). These eight constructs were categorized as (1) exogenous variables (mobile self-efficacy and trust), (2) endogenous variables (PE, EE, perceived enjoyment, satisfaction, and BI), and (3) a moderator variable (PR). Each construct is measured by multiple items. To quantify the constructs, a 5-point Likert scale was adopted to score questionnaire responses. The Likert scale consisted of five answer options ranging from “*strongly disagree*” (mapped to number 1) to “*strongly agree*” (mapped to number 5). The second section contained demographic information presented on a nominal scale. The questionnaire collected basic information about respondent characteristics, including age, gender, school, and grade.

The instrument (i.e., EE, PE, BI, mobile self-efficacy, perceived enjoyment, satisfaction, trust, and PR) was developed after a thorough review of studies related to the UTAUT model. Following MacKenzie et al. (2011) and the development procedures suggested by DeVellis (2003), standard psychometric scales were developed. The main constructs of the UTAUT model (i.e., EE, PE, and BI) were adopted from measurement constructs developed in related studies (Venkatesh et al., 2003; Cimperman et al., 2016; Šumak and Šorgo, 2016; Hoque and Sorwar, 2017; Khalilzadeh et al., 2017; Šumak et al., 2017). The EE measure contained five items, PE had four items, and BI had three items. Students’ mobile self-efficacy in m-learning was measured based on three items from related studies (Bandura, 1986; Ozturk et al., 2016; Nikou and Economides, 2017); perceived enjoyment contained three items (Venkatesh, 2000; Park et al., 2012; Chang et al., 2017; Tsai et al., 2018), satisfaction had five items (Maillet et al., 2015; DeLone and McLean, 2016), trust contained five items (Arpaci, 2016; Alalwan et al., 2017; Kabra et al., 2017; Khalilzadeh et al., 2017) and PR had three items (Hanafizadeh et al., 2014; Martins et al., 2014; Alalwan et al., 2018). Details on the questionnaire used are shown in Appendix Table A1. To improve the questionnaire’s validity, we conducted a pilot study prior to the actual test. The main objective of the pilot study was to empirically validate the reliability of the questionnaire by checking the accuracy and precision of all measurement items (Hair et al., 2010). For each construct, reliability was checked based on Cronbach’s alpha, for which the threshold was set to 0.7 (Hair et al., 2010). In the pilot test, we received 122 complete responses from students at two universities in Taichung, Taiwan. The reliability scores, which were based on the Cronbach’s alpha scores, ranged from 0.758 for PR to 0.898 for satisfaction. The results indicated that the Cronbach’s alpha values for all variables exceeded 0.7. After the appropriate level of reliability had been confirmed for all measurement items, the final questionnaire proved reliable and usable.

### Participants

Empirical data were collected using a cross-sectional survey. We recruited 2,000 students from ten universities (including general universities and universities of science and technology) in Taiwan. Two hundred students were randomly selected from each sample university. All participating students had experience of using mobile devices for personal learning. To maximize the survey response rate, we recruited a contact person at each selected school to manage the questionnaire distribution process. The study ethics procedures were executed according to the 1964 Helsinki declaration and its later amendments or comparable ethical standards and the ethical norms of the Taiwan Ministry of Science and Technology do not require ethical external approval. This exemption was because the data was anonymous and there is no way for readers to be able to identify the participants. There are no name lists that correspond to the respondents of questionnaire and the names of the participating universities were not mentioned. All subjects were informed about the research and all participants include in the study provided informed consent. All respondents were volunteers and were assured that their responses would remain anonymous, their confidentiality would be maintained, and their answers would be used only for research purposes. It took the participants 15–20 min to complete the questionnaire. A total of 1,736 questionnaires were collected and prescreened based on the respondents’ m-learning experiences. Subsequently, 174 incomplete responses were rejected, leaving 1,562 valid questionnaires for formal data analysis.

The demographic characteristics of the respondents are presented in Table 1. The data revealed that the mean age of the participants was 19.6 years (standard deviation: 1.4 years). Approximately two-thirds of the participants were women (67.0%). In addition, 37.1% of the sample were from management colleges. Approximately 45% of the participants were in their first year in college.

**TABLE 1:** Profile of Respondents (*N* = 1,562).

**Demographics/ Level**	***N***	**Percentage**	**Demographics/ Level**	***N***	**Percentage**
**Gender**			**Year in college**		
Male	516	33.0	First	702	44.9
Female	1046	67.0	Second	444	28.4
**College**			Third	225	14.4
College of Science and Engineering	288	18.4	Fourth	191	12.2
College of Humanities and Social Sciences	335	21.4			
College of Design	359	23.0			
College of Management	580	37.1			

## Results

### Data Analysis

Partial least squares (PLS) regression is one of the most commonly adopted structural equation modeling (SEM) techniques used to validate structured data. PLS regression is especially effective for data analysis during the early stages of theory development when the theoretical model and its measures are not yet complete (Tsang, 2002). The PLS model analyzes and interprets the reliability and validity of (1) the measurement model and (2) the structural model. In this study, PLS regression was used to perform bootstrapping for our research model and to test and validate the proposed model and the relationships among the hypothesized constructs.

### Measurement Model Evaluation

The measurement model was assessed by examining the internal reliability, convergent validity (CV), and discriminant validity (DV). The internal reliability was evaluated by examining the Cronbach’s alpha and composite reliability (CR) values for all constructs. CV was assessed by measuring the average variance extracted (AVE). Accordingly, the three most commonly used evaluation indicators were selected (Fornell and Larcker, 1981; Chin, 1998; Jöreskog and Sörbom, 2005; Hair et al., 2010; Bagozzi and Yi, 2012), include: Cronbach’s alpha, composite reliability (CR), and average variance extracted (AVE). The item loading range, Cronbach’s alpha, AVE, and CR results are presented in Table 2.

**TABLE 2:** Construct Reliability Results.

**Construct**	**No. of items**	**Item loading**	**Cronbach’s α**	**AVE**	**CR**
Perceived Enjoyment (PEN)	3	0.79–0.85	0.76	0.675	0.861
Effort Expectancy (EE)	5	0.73–0.80	0.82	0.584	0.875
Performance Expectancy (PE)	4	0.70–0.82	0.77	0.589	0.851
Satisfaction (SAT)	5	0.84–0.88	0.90	0.722	0.928
Trust (TRU)	5	0.77–0.88	0.89	0.694	0.919
Mobile Self-efficacy (M-SE)	3	0.85–0.88	0.82	0.736	0.893
Perceived Risk (PR)	3	0.68–0.96	0.70	0.629	0.761
Behavioral Intention (BI)	3	0.86–0.89	0.85	0.772	0.910

*AVE, Average Variance Extracted; CR, Composite Reliability.*

In Table 2, the estimated construct loadings range from 0.681 to 0.960, and thus are higher than the recommended levels (Hair et al., 2010). Construct reliability indicates how well a construct is measured by its items, and can be assessed based on Cronbach’s alpha and CR. The Cronbach’s alpha values ranged from 0.70 for PR to 0.90 for satisfaction, and CR values ranged from 0.761 for PR to 0.928 for satisfaction. For both measures, all constructs exceeded the recommended cutoff of 0.7 (Fornell and Larcker, 1981; Hair et al., 2010), thereby suggesting high internal reliability. Table 2 reveals that the estimated latent construct factor loadings ranged from 0.68 to 0.96 and were statistically significant (*p* < 0.05). The AVE ranged from 0.584 (EE) to 0.772 (BI) and was greater than 0.5 for each construct (Fornell and Larcker, 1981), thereby indicating CV.

To evaluate the DV, the square root of the AVE of each latent construct was compared with its interconstruct correlation. The square root of the AVE of a construct should be greater than its correlations with other constructs to achieve satisfactory DV (Fornell and Larcker, 1981; Hair et al., 2016). Additionally, the diagonal values should be higher than the off-diagonal values in the corresponding columns and rows (Henseler et al., 2009). As shown in Table 3, for each construct, the square root of the AVE (shown diagonally with bold values) exceeded the inter-construct correlations, thereby indicating an appropriate level of DV.

**TABLE 3:** Correlation matrix and square root of the AVE.

**Construct**	**Mean**	**SD**	**PEN**	**EE**	**PE**	**SAT**	**TRU**	**M-SE**	**PR**	**BI**
PEN	3.47	0.70	**0.82**							
EE	3.54	0.65	0.46^*^	**0.76**						
PE	3.63	0.63	0.47^*^	0.57^*^	**0.77**					
SAT	3.41	0.68	0.54^*^	0.52^*^	0.50^*^	**0.85**				
TRU	3.25	0.69	0.45^*^	0.57^*^	0.43^*^	0.61^*^	**0.83**			
M-SE	3.93	0.70	0.51^*^	0.46^*^	0.46^*^	0.37^*^	0.25^*^	**0.86**		
PR	2.09	0.71	−0.20^*^	−0.12^*^	−0.22^*^	−0.05^*^	−0.05^*^	−0.29^*^	**0.79**	
BI	3.34	0.77	0.57^*^	0.47^*^	0.49^*^	0.63^*^	0.51^*^	0.40^*^	−0.08^*^	**0.88**

SD, Standard deviation; Bolded values on the diagonal are the square root of the AVE. Values on the off-diagonal represent inter-construct correlations. PEN, Perceived enjoyment; EE, Effort expectancy; PE, Performance expectancy; SAT, Satisfaction; TRU, Trust; M-SE, Mobile self-efficacy; PR, Perceived risk; BI, Behavioral intention. ^*^p < 0.05.

### Statistical Analysis and Hypotheses Testing

**FIGURE 3:**
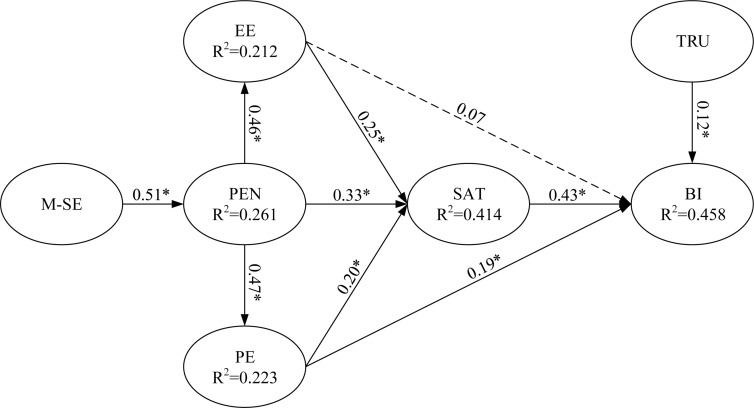
Path coefficients for the research model (excluding moderator main effect). Value on path: standardized coefficients (β), *R*^2^: Coefficient of determination and ^*^p < 0.05.

**FIGURE 4:**
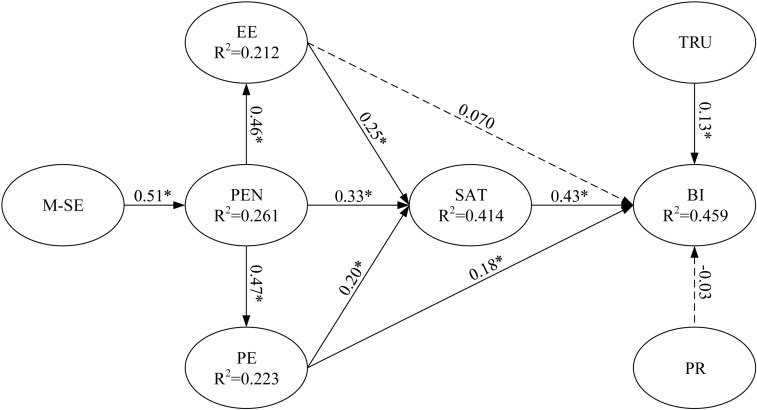
Path coefficients for the research model (including moderator main effect). Value on path: standardized coefficients (β), *R*^2^: Coefficient of determination and ^*^p < 0.05.

Partial least squares regression was used to test the main effects of EE and PE and the moderating effect of PR on BI to use m-learning (Figures 3, 4, respectively). For example, to test the moderating effect, PE (predictor) and PR (moderator) were multiplied to create an interaction construct (PE × PR) for predicting BI to use m-learning.

Regarding the overall quality of the research model, the SEM procedure based on PLS regression was applied to analyze the goodness of fit (GoF), path coefficients, and coefficient of determination (*R*^2^). The GoF (0 < GoF < 1) is considered the geometric mean of the average commonality and average *R*^2^ value. To measure the GoF, this study used the equation employed by Alolah et al. (2014): $\mathrm{GoF}=\sqrt{\bar{A\,V\,E}\times {\bar{R}}^{2}}$. In our study, the GoF value was 0.502, which exceeded the 0.36 benchmark suggested by Tenenhaus et al. (2005). Thus, the proposed model had good overall fit, indicating that it performed well compared with the aforementioned baseline values.

This study tested the relationships between dependent and independent variables by using the path coefficient (β) and *t* statistics. By using PLS regression to estimate the path relationship of each pair of research constructs, among all eight path relationships, we revealed that seven assumptions attained significance. Bootstrapping resampling was performed to test the significance of the path coefficients in the inner model (number of iterations: 1000).

**FIGURE 5:**
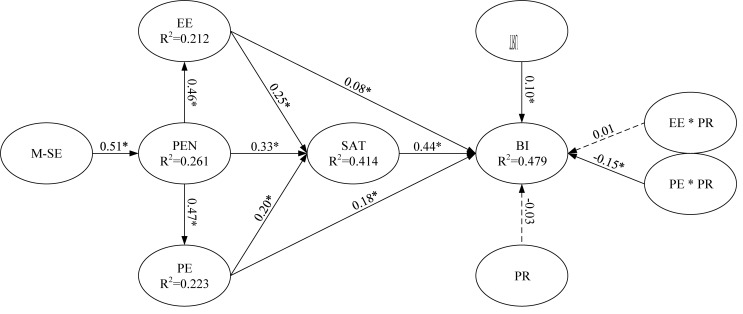
Path coefficients for the research model (including interaction effect). Value on path: standardized coefficients (β), *R*^2^: Coefficient of determination and ^*^p < 0.05.

To verify the hypotheses and moderating effects, the moderator analysis method proposed by Baron and Kenny (1986) was followed. The empirical analysis determined the moderating roles of PR based on the significance of the interaction terms in Model 3. Among the two hypothesized moderating effects, M1 was non-significant; that is, PR did not have moderating effect on the relationship between EE and BI. However; PR negatively moderated the relationship between PE and BI in relation to m-learning use (M2: β = −0.15, *p* < 0.05); this finding indicates that M2 was significant. These additional analyses provided support for the moderation pattern presented in our model. Figure 5 provides all results of the moderation analysis, including the structural path estimates and explained variances. Consistent with M2, PE and PR had a negative effect on BI to use m-learning. Specifically, we revealed that PE and BI related to m-learning increased with a decrease in PR.

Regarding the components of the UTAUT model, EE and PE had significantly positive effects on BI to use m-learning (β = 0.08 and 0.18, respectively, *p* < 0.05). Therefore, Hypotheses 1 and 2 were supported. In addition, satisfaction and trust had significant positive effects on BI (β = 0.44 and 0.10, respectively, *p* < 0.05), thereby supporting Hypotheses 3 and 4. EE, PE, and perceived enjoyment were all crucial antecedents of satisfaction (β = 0.25, 0.20, and 0.33, respectively, *p* < 0.05). The results for the prediction of satisfaction were consistent with the EE, PE, and perceived enjoyment hypotheses adapted to the context; thus, Hypotheses 5, 6, and 7 were supported. Perceived enjoyment was a significant determinant of EE and PE (β = 0.46 and 0.47, respectively), thereby supporting Hypotheses 8 and 9. Finally, mobile self-efficacy was a significant determinant of perceived enjoyment (β = 0.51, *p* < 0.05), and thus Hypothesis 10 was supported.

Figure 5 presents the explanatory power. The model explained a substantial portion of the variance in all endogenous variables: EE (21.2%), PE (22.3%), perceived enjoyment (26.1%), satisfaction (41.4%), and BI (47.9%). Falk and Miller (1992) asserted that the coefficient of determination (R^2^) should be higher than 0.10; all the endogenous variables in our study satisfied this requirement. However, a substantial portion of unexplained variances indicated that other key factors beyond the scope of this study could be incorporated to improve the explanatory power of the endogenous variables. In summary, the model employed in this study explained a considerable number of variations in the endogenous variables. The endogenous variables exhibited strong explanatory power for these variations, thereby indicating the stability and robustness of the model. All estimated and standardized path coefficients (significant paths are indicated with asterisks) are illustrated in Figure 5.

## Discussion

The purpose of this study was to identify factors that affect university students’ BIs to use m-learning. The research model presented in this paper is unique in its integration of perceived enjoyment, mobile self-efficacy, satisfaction, trust, PR, and BI into the UTAUT model to evaluate the determinants of users’ BIs toward m-learning. This model examined whether PE, EE, and PR moderated and predicted BI. The results of a cross-sectional online survey of 1,562 participants demonstrated that the fundamental determinants of BI were, in order of relevance, satisfaction, PE, trust, and EE. In addition, the results revealed positive influences of perceived enjoyment, PE, and EE on satisfaction. The negative moderating role of PR on the relationship between PE and BI was also revealed. An interpretation of the results based on the empirical findings is presented as follows.

The research model explained 47.9% of the variance in BI. The most crucial factors that influenced BI were satisfaction, PE, trust, and EE. Satisfaction and trust had direct effects on BI to use m-learning; this was consistent with the findings of another study (Koufaris and Hampton-Sosa, 2004; DeLone and McLean, 2016; Kabra et al., 2017). Therefore, satisfaction and trust are crucial predictors of individuals’ BIs to adopt ISs or IT. The Taiwanese government has been promoting online learning in primary and secondary education since 1996 to cultivate literacy in IT and improve students’ international competitiveness. Consequently, most current students have been receiving IT education since the third or fourth grade; this policy has equipped students with the basic ability to adapt to changes in technology. In this study, all participating students had received IT education at elementary school. As technology continues to evolve, students learn not only through face-to-face teaching and e-learning systems but also increasingly through m-learning. Many students have realized the advantages of e-learning and m-learning. In particular, m-learning fits students’ requirements to learn without time and space limitations. In the contemporary world, m-learning is relatively accessible, thereby providing a favorable m-learning environment and promoting students’ BIs. Thus, the higher students’ satisfaction and trust toward m-learning, the higher are their BIs. The findings of the study confirmed that PE and EE had significantly positive effects on BI; this was in accordance with the findings of other studies (Venkatesh et al., 2003; Šumak and Šorgo, 2016; Hoque and Sorwar, 2017; Khalilzadeh et al., 2017; Šumak et al., 2017). In addition, the results of our analysis highlighted the fundamental role of PE. We revealed that PE, alongside perceived enjoyment and EE, is positively associated with satisfaction with m-learning. This indicates that perceived enjoyment had a significantly positive effect on satisfaction with m-learning, which corresponds with the findings of Shiau and Luo (2013). Furthermore, we demonstrated that the effects of PE and EE on satisfaction with m-learning were significant and positive; this is similar to the findings of Maillet et al. (2015). Based on the findings of the present study, m-learning is an increasingly crucial method of learning for students. When students find m-learning engaging and easy to use and consider it to improve their learning performance and effectiveness, their satisfaction toward m-learning and their BIs toward using it are enhanced. Therefore, regarding the future development of m-learning, schools and other educational institutions are recommended to provide online forums for learners to communicate and share what they have learned. This measure could promote diversity with respect to m-learning and increase students’ satisfaction and BIs to use it.

Most related studies (Sánchez-Prieto et al., 2016; Chang et al., 2017; Tsai et al., 2018) have argued that perceived enjoyment is a crucial external factor that significantly affects the PU and PEOU of m-learning. However, to our knowledge, no studies have investigated the effects of perceived enjoyment on PE and EE; thus, a theoretical foundation is yet to be built. The findings of this study demonstrated that perceived enjoyment significantly influenced PE and EE. Therefore, perceived enjoyment is a key external variable in the UTAUT model. In addition, no study has examined the possible effect of mobile self-efficacy on perceived enjoyment. The result obtained in the present study indicated that mobile self-efficacy had a significantly positive effect on perceived enjoyment. We expanded the use of mobile self-efficacy and perceived enjoyment. With the popularity of the Internet and mobile devices for various uses (e.g., mobile payments, banking, and mobile health), university students have high mobile self-efficacy and gain enjoyment from using their mobile devices. As m-learning becomes an increasingly dominant method of learning, students’ enjoyment of it is expected to increase. Students not only find m-learning easy to use but also acknowledge the importance of learning.

In this study, PR was tested as a moderator; the results revealed that it significantly and negatively moderated the relationship between PE and BI. This significant relationship indicated that (1) PR as a moderating variable provided a robust basis for our hypotheses, and (2) PR was a critical moderating variable for m-learning usage in our extended UTAUT model. However, PR did not have a moderating effect on the relationship between EE and BI. According to our findings, if university students perceive m-learning as easy to use, their level of PR plays no fundamental role in the decision to use it. However, the relationship between PR and BI was non-significant; this finding differs from that obtained by Alalwan et al. (2018). Based on our findings, in addition to their basic understanding of m-learning, students are aware of solutions (e.g., system instruction, FAQs, and online forums) to potential risks and problems and that the privacy and safety of systems have been improved in recent years. These factors can lower students’ PR. Therefore, PR did not have a significant influence on BI. Notably, when using m-learning, students worry about problems that could hinder their learning (e.g., Internet stability and whether they have successfully uploaded assignments and updated data), thereby increasing PR and reducing BI. Thus, schools and system developers should establish a feedback mechanism through which students can find out whether their assignments were successfully uploaded to the system7; this measure could lower PR and increase BI.

## Limitations and Future Research

This study had several limitations that could be addressed in future studies. First, the results were based on university students, and thus could benefit from comparison with results obtained from the same model aimed at students from a wider variety of educational levels (e.g., senior and vocational high school students). Second, this study was cross-sectional in nature and conducted within a short period. Students’ perceptions of EE, PE, satisfaction, trust, and BI toward m-learning can change over time as new knowledge and experiences are accumulated. Therefore, future studies could employ a longitudinal design to obtain more accurate findings from a specific group. Finally, although the moderator of this study was PR, other variables such as system quality, trust, and mobile information literacy may also moderate the relationship between BI and another factor/variable. Thus, these variables should be considered as moderators in future studies. Finally, this study used a self-reported questionnaire as the research tool. In a questionnaire, when answering questions, interviewees might not express their true opinions, and this could lead to errors in the results. This problem should be handled cautiously when interpreting research data.

## Conclusion

This study developed a novel integrative model to explain the determinants of university students’ BIs toward using m-learning at an individual level. A conceptual model was built based on the UTAUT model in to extend this adequately validated framework by incorporating five additional predictor variables (i.e., mobile self-efficacy, perceived enjoyment, satisfaction, trust, and PR). Data were collected from 1,562 participants with experience in using m-learning. The results revealed that the model had high internal consistency and reliability, thereby indicating that the proposed model possesses substantial explanatory power. This study revealed that satisfaction is a key factor that significantly influences university student’s BIs toward using m-learning. In addition, the results revealed positive influences of PE, trust, and EE on BI. Students’ perceived enjoyment was a key factor that affected PE, EE, and satisfaction. Mobile self-efficacy had a significant positive effect on perceived enjoyment. Finally, PE and PR had a negative interaction effect on BI to use m-learning. Determining what motivates use of new technologies can improve learning quality and boost pedagogical and instructional uses of said technologies. The findings of this study could be of value for decision-makers in educational institutions.

## Author Contributions

The author confirms being the sole contributor of this work and has approved it for publication.

## Conflict of Interest Statement

The author declares that the research was conducted in the absence of any commercial or financial relationships that could be construed as a potential conflict of interest.

## Appendix

**TABLE A1:** Measurement Items.

**Constructs**	**Items**	**Mean**	**SD**
**Effort Expectancy**	Learning how to use mobile learning is easy for me.	3.58	0.84
	My interaction with the mobile learning would be clear and understandable.	3.47	0.91
	I find mobile learning easy to use.	3.71	0.80
	It is easy for me to become skilful at using mobile learning.	3.62	0.82
	I would find it easy to get the mobile learning to do what I want it to do.	3.33	0.89
**Performance Expectancy**	Using the mobile learning would improve my learning performance.	3.61	0.79
	Using mobile learning increases my chances of achieving learn that are important to me.	3.55	0.83
	Using the mobile learning would allow me to accomplish learning tasks more quickly.	3.72	0.86
	Using the mobile learning would enhance my effectiveness in learning.	3.65	0.79
**Perceived Enjoyment**	I find using mobile learning enjoyable.	3.36	0.90
	The actual process of using the mobile learning is pleasant.	3.49	0.81
	I have fun using the mobile learning.	3.56	0.83
**Satisfaction**	I was very content with mobile learning.	3.44	0.77
	I was very pleased with mobile learning.	3.36	0.77
	I was satisfied with mobile learning efficiency.	3.38	0.79
	I felt delighted with mobile learning.	3.43	0.86
	Overall, I was satisfied with mobile learning.	3.44	0.81
**Trust**	I believe that mobile learning is trustworthy.	3.30	0.78
	I trust in mobile learning.	3.30	0.79
	I do not doubt the honesty of mobile learning.	3.30	0.85
	Even if not monitored, I would trust mobile learning to do the job right.	3.24	0.79
	Mobile learning has the ability to fulfill its task.	3.10	0.93
**Mobile Self-efficacy**	I am confident of using the mobile learning even if there is no one around to show me how to do it.	3.86	0.84
	I am confident of using the mobile learning even if I have never used such a system before.	3.96	0.79
	I am confident of using the mobile learning even if I have only the software manuals for reference.	3.97	0.81
**Perceived Risk**	I think using mobile learning puts my privacy at risk.	3.99	0.86
	Using mobile learning exposes me to an overall risk.	3.99	0.84
	Using mobile learning will not fit well with my self-image.	3.83	0.87
**Behavioral Intention**	Assuming I had access to the mobile learning, I intend to use it.	3.16	0.92
	Given that I had access to the mobile learning, I predict that I would use it.	3.46	0.82
	I plan to use the mobile learning in the future.	3.39	0.87
